# Postgraduate training for family medicine in a rural district hospital in South Africa: Appropriateness and sufficiency of theatre procedures as a sentinel indicator

**DOI:** 10.4102/phcfm.v8i1.1106

**Published:** 2016-06-30

**Authors:** Dawie Du Plessis, Paul Alfred Kapp, Louis S. Jenkins, Laurel Giddy

**Affiliations:** 1 Division of Family Medicine and Primary Care, University of Stellenbosch, South Africa; 2 Knysna and Bitou sub-districts, Western Cape, South Africa; 3 Family Medicine, George Hospital, Eden District, Western Cape, South Africa

## Abstract

**Background:**

Since 2007, the postgraduate training of family physicians for South African district hospitals has been formalised. This training differs from European and North American programmes as up to 30% of the skills needed rely on district hospital surgical, obstetrics and anaesthetics procedures, particularly in rural areas, as outlined in the national unit standards. The aim of this study was to evaluate the appropriateness and sufficiency of learning opportunities for these skills in a rural district hospital.

**Methods:**

A descriptive, cross-sectional study was undertaken of the number and type of procedures performed in theatre for a 1-year period and compared with the required procedural skills stipulated in the national unit standards. Descriptive statistical analyses were used to analyse categorical data.

**Results:**

Three thousand seven hundred and forty-one procedures were performed during the study period. Anaesthesia was the most common procedure, followed by Caesarean section. There were adequate opportunities for teaching most core skills.

**Conclusions:**

Sufficient and appropriate learning opportunities exist for postgraduate family medicine training in all the core skills performed in a theatre according to the national unit standards.

## Background

Since the recognition of family medicine as a speciality by the Colleges of Medicine of South Africa and the Health Professions Council of South Africa in 2007, national core competencies and minimum skills levels for the discipline have been established.^1^ This is in keeping with international norms to ensure uniformity in the training and expected competencies of specialist family physicians.^2,3^

The objectives of postgraduate family medicine training have been well described, namely that ‘There is to be a compulsory, full-time four-year training programme for registrars in Family Medicine which should result in a well-trained cohort of Family Physicians suitable to staff Community Health Centres (CHC) and Primary Care Hospitals in the future’.^4^

As early as 1987, it was identified that healthcare in Africa required the training of a ‘district hospital doctor’ who has a wide range of procedural skills.^5^ More recently, in the local context, there have been extensive reports on the skills and knowledge requirements of doctors who work in district hospitals in the Western Cape.^6,7,8^

The South African National Department of Health published the expected package of care required for a district hospital in a 2002 policy document. This was supplemented in 2009 by the Western Cape District Package of Care.^9,10^ The required skills in the national and provincial district packages of care are similar to the skills and competencies expected of a family physician.^1^

Family physicians and medical officers (MOs) in district hospitals need to be competent in a range of surgical and anaesthetic procedures, and there is a need for ongoing training.^5,6,7,8^ Initially, the postgraduate training programme offered by Stellenbosch University (SU) had a 2-year period of rotations through the major disciplines in a regional hospital, with training largely supervised by consultants from outside the speciality of family medicine and 2 years spent in a district hospital and CHCs. More recently, there has been a shift towards offering most of the training at the district hospital and CHCs (3–4 years), with focussed exposures in level 2 facilities should the need arise (0–1 year).^11^ Family medicine educators in South Africa consider it imperative ‘That training would take place in CHC, Level 1 (district or primary care orientated) hospitals and where necessary, in Level 2 (regional hospitals)’.^4^ Postgraduate training within the primary care context ‘provides opportunity for the registrar to gain experience in community-oriented primary care, ambulatory care, the care of families, continuity of care, and promotive, preventive and rehabilitative health care’.^12^

Knysna District hospital is a 96-bed provincial hospital situated in the Eden District of the Western Cape Province of South Africa. It provides comprehensive hospital- and clinic-based primary care services to a population of roughly 120 000 people who reside within its drainage area.^13,14^ The doctors who provide the service are led by two family physicians and comprise a mixture of MOs of varying experience and community service MOs. Some surgical services that are appropriate to level 2 and level 3 care are performed by outreaching specialists in their respective fields. The hospital is a sought-after training site among family medicine registrars and has a 100% pass rate including two cum laude students. Currently, there are three registrar posts for postgraduate family medicine training at the hospital. The aim of this study was to evaluate the appropriateness and sufficiency of learning opportunities for the nationally expected skills in surgery, obstetrics and anaesthetics in a rural district hospital, particularly Knysna hospital.

## Methods

The study was done in the theatres in Knysna District hospital in the Eden District of the Western Cape Province of South Africa. All surgical and anaesthetic procedures done between 1 February 2011 and 31 January 2012 were recorded from the entries made into the theatre record. The study was designed for a 12-month period in order to incorporate any seasonal changes and other factors to try to ensure that it will be representative. When counting the procedures, every subentry was counted as a procedure. For instance, a Caesarean section and tubal ligation was counted as three procedures: Anaesthetic (spinal/general), Caesarean section and Tubal ligation. Data were recorded in an Excel 2007™ spreadsheet. Skills and procedures were used as interchangeable terms, conveying similar meaning. Opportunities for teaching family medicine registrars procedural skills were identified and compared with the national skills list for family medicine training. Each procedure was counted and tabulated according to the experience level of the doctor who performed the procedure. Doctors with a minimum of 5 years’ experience or a specialist qualification were identified as teachers and all procedures performed by a teacher were categorised as a learning opportunity. Five years’ experience was decided on as an arbitrary level of experience in order to ensure that the teacher would have sufficient experience to enable skills transfer.

Every procedure on the skills list that was done in theatre was tabulated. The national skills list differentiates procedures as either core, supervised or elective skills and the same definitions were used in this study (see Table 1).^1^

**TABLE 1:** Definitions as encompassed in the agreed national skills list.

Term	Definition
Core skills	Skills that should be performed independently at the end of training
Supervised skills	Skills that should be performed under supervision during training
Elective skills	Skills that can be taught in specific programmes but are not required as part of national training. Other elective skills not listed here may also be relevant to individual students/settings

*Source*: From Couper I, Mash B. Obtaining consensus on core clinical skills for training in family medicine. S Afr Fam Pract. 2008;50(6):69–73

The literature suggests that a skill needs to be performed 15–25 times by a trainee in order to obtain proficiency.^15,16,17,18^ A value of 20 was used for the purpose of this study. Given the 4-year duration of the family medicine training programme, all procedures that occurred as learning opportunities five times during the year under review were regarded as adequate to enable the training of one registrar to become proficient in 4 years provided the registrar is given priority in accessing the learning opportunity. Some of the procedures performed were not applicable to family medicine training and were excluded.

All procedures performed outside the theatre in the wards, out-patient department (OPD) or emergency centre (EC) were excluded. The study focused only on the availability of learning opportunities and made no attempt to determine whether these opportunities were utilised.

### Ethical considerations

Ethical approval for the research was obtained from the Health Research Ethics Committee at SU (S13/07/128) and permission to use the heath facility for research from the Western Cape Department of Health.

## Results

**FIGURE 1:**
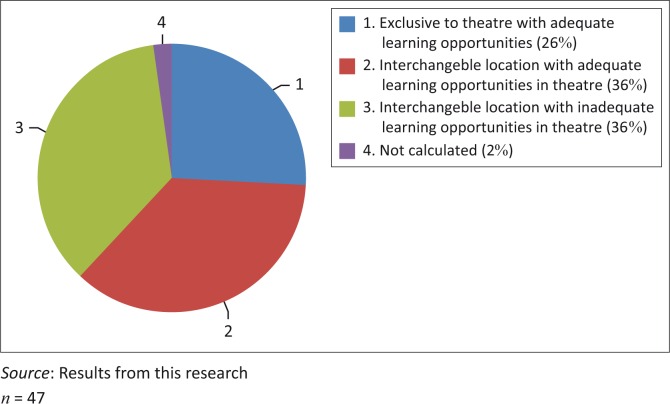
Core procedures performed in theatre.

A total of 3741 surgical, obstetrics and anaesthetic procedures were done. Of these, 3737 were identifiable and 4 were unknown or illegible in the theatre record. One hundred and twenty-six different procedures were recorded with the frequency varying from 1 to 1202 per year. The different procedures are listed according to their classification as either core, supervised or elective skills (see Appendix 1, 2 and 3, Figure 1 and Tables 2, 3 and 4).

**TABLE 2:** Core skills.

Exclusive theatre procedures for which there were sufficient learning opportunities	Procedures that could be done inside or outside theatre for which there were sufficient learning opportunities in theatre	Procedures that could be done inside or outside theatre for which there were no sufficient learning opportunities in theatre
Caesarean section Evacuation of uterus Tubal ligation Debridement of wounds or burns Check Boyle’s machine General anaesthetic Inhalation induction Intravenous induction Monitor patient during anaesthesia Recover patient in a recovery room Reverse muscle relaxation Set airflows – Magill’s, circle T-piece Spinal anaesthetic	Incision and drainage of perianal haematoma Penile block Circumcision Excision of sebaceous cyst (other lumps or bumps), Cauterisation or cryotherapy Skin biopsy (excision or punch) Inserting intrauterine contraceptive device Dilatation and curettage Drainage Bartholin abscess or cyst Incision and drainage of abscess Closed reduction of fracture Excise a ganglion Administering oxygen Control of airway and mask ventilation Endotracheal intubation and ventilation of patient Ketamine anaesthesia Ventilation of a patient with a Bag-Mask-Valve device	Lymph node excision biopsy Proctoscopy Drain a hydrocoele Insert a suprapubic catheter Incision and drainage of chalazion Suture an eyelid Remove foreign body ear Remove foreign body nose Epistaxis (Cautery and packing) Drain peritonsillar abscess Manual removal of placenta Suturing third-degree tear Reduce elbow dislocation Reduce hip dislocation Reduce radial head dislocation Reduce shoulder dislocation Ring block (digit)

*Source*: Results from this research

**TABLE 3:** Supervised skills.

Exclusive theatre procedures for which there were sufficient learning opportunities	Exclusive theatre procedures for which there were no sufficient learning opportunities	Procedures that could be done inside or outside theatre for which there were no sufficient learning opportunities in theatre
Appendectomy Vasectomy Skin graft Laparotomy for ectopic pregnancy Termination of pregnancy	Hydrocoelectomy Amputation of fingers Fasciotomy Biers’ block Brachial block Epidural anaesthesia	Pleural biopsy Reduction of nose fracture Injection of keloids Phenol ablation of ingrown toenail Relieve cardiac tamponade. Tracheostomy Debridement of open fractures

*Source*: Results from this research

**TABLE 4:** Elective skills.

Exclusive theatre procedures for which there were sufficient learning opportunities	Procedures that could be done inside or outside theatre for which there were sufficient learning opportunities in theatre
Hernia repair Bilateral sub-capsular orchidectomy Cataract removal Tonsillectomy and adenoidectomy Colposcopy Hysterectomy Loop electrosurgical excision procedure for cervix Explorative laparotomy (for trauma or bowel obstruction) Open reductions, pins and screws of fractures Repair nerves and tendons	Prostate biopsy Dental extraction

*Source*: Results from this research

## Discussion

A wide spectrum of procedures (126) was performed during the study period. General anaesthesia was the procedure performed most often (1202 times). This was expected because most other procedures would need anaesthesia in order to make their performance possible. Of the surgical procedures, obstetric and gynaecological procedures dominated. Some of the procedures performed occurred frequently and presented high volumes of learning opportunities so that proficiency can be taught within 1 year. These relatively common procedures were mostly those procedures designated for district (level 1) facilities, for example, general anaesthetics and Caesarean sections.

Of the 47 procedures considered core skills that were done in theatre, 34 (72%) could also be done in the wards, OPD or the EC. This interchangeability of the core skills between theatre and elsewhere illustrates the flexibility required of family physicians. This is congruent with the African understanding that family physicians must be competent to work in both the ambulatory primary care as well as the district hospital environment.^19^ Twenty-nine of the 47 procedures occurred often enough as learning opportunities in theatre to enable training of registrars, depending on the work schedules and after-hours call rosters of the registrars. One skill, checking a Boyle’s machine, was not counted and could not be commented on, although it was assumed to be performed at least on a daily basis. Seventeen core procedures that were performed both in theatre and other procedure rooms were not performed often enough in theatre to be classified as sufficient learning opportunities. There was a lack of data with regards to how often these procedures were done outside theatre and fell outside the scope of this research.

There were 18 supervised skills that happened in theatre. The definition of supervised skills is ambiguous concerning the level of proficiency and independence the registrar should display in performing the procedure. If the requirement is for the registrar to only perform the procedure once with expert guidance, as the definition suggests, a single learning opportunity would suffice and the number of procedures that had adequate learning opportunities will increase. We intended to explore to what extent registrars can be taught proficiency; therefore, we have not altered our definition of adequate learning opportunities. During the year in review, 5 of the 18 procedures potentially had sufficient learning opportunities to enable registrars to become proficient in a 4-year training period. Of the remaining 13 procedures, 6 were exclusive to theatre but did not occur often enough for a registrar to become proficient to perform the procedure independently. Seven of the procedures could be performed in theatre or elsewhere. It is acknowledged that these potential learning opportunities would depend on the availability of registrars, the number of registrars at any one time in the hospital and other system factors such as staff leave, equipment and patient dynamics.

Elective skills, by definition, are skills that a registrar chooses to learn for personal reasons or because there is a local need for a skill. If a procedure is repeated often enough to enable training, there is presumably a need for that skill in the local context. The availability of sufficient learning opportunities can therefore be used to inform a decision about learning a skill.

## Limitations

This study quantified learning opportunities inside theatre and excluded procedures done outside theatre. Therefore, it was not possible to comment on the hospital as a whole for postgraduate training opportunities. However, focusing on theatre allowed for detailed and accurate data analysis, serving as a sentinel indicator for the rest of the hospital.

## Conclusion

The aim of this study was to evaluate the appropriateness and sufficiency of learning opportunities for the nationally expected skills in surgery, obstetrics and anaesthetics in a rural district hospital, particularly Knysna hospital theatres. This small, rural district hospital provided adequate training opportunities for most of the core procedural skills expected for postgraduate family medicine training in South Africa. Most of the procedures considered supervised skills did not occur often enough to enable training. Elective skills that are directed by local service needs can be taught adequately. These results suggest that district hospitals in similar settings in South Africa are adequate for postgraduate family medicine training.

## Appendix 1

**Table:**

Core clinical skills	More than 20 learning opportunities	More than 5 learning opportunities	Insufficient learning opportunities	Procedures that happen inside theatre and elsewhere
Lymph node excision biopsy			4	*
Incision and drainage of perianal haematoma		9		*
Proctoscopy			0	*
Penile block	78			*
Circumcision	101			*
Drain a hydrocoele			0	*
Insert a suprapubic catheter			2	*
Incision and drainage of chalazion			3	*
Suture an eyelid			0	*
Remove foreign body ear			3	*
Remove foreign body nose			0	*
Epistaxis (cautery and packing)			0	*
Drain peritonsillar abscess			0	*
Excise sebaceous cyst (other lumps/bumps)		10		*
Cryotherapy/cauterisation		6		*
Skin biopsy	29			*
Caesarean section	150			
Evacuation of uterus	58			
Manual removal of retained placenta			2	*
Repair of third-degree tear			3	*
Intrauterine contraceptive device		5		*
Dilatation and curettage		6		*
Drainage Bartholin abscess or cyst		10		*
Tubal ligation	128			
Debride wounds or burns		17		
Incision and drainage of abscess	23			*
Closed reduction of fractures		6		*
Reduce elbow dislocation			0	*
Reduce hip dislocation			0	*
Reduce radial head dislocation			0	*
Reduce shoulder dislocation			2	*
Excise a ganglion		12		*
Ring block			0	*
Administer oxygen	1014			*
Check Boyle’s machine	Not counted			
Control airway, mask ventilation	1014			*
General anaesthetic	1014			
Inhalation induction	134			
Intravenous induction	880			
Intubation and ventilation of patient^#^	500			*
Ketamine anaesthesia	64			*
Monitor patient during anaesthetic	1240			
Recover patient in recovery room	1240			
Reverse muscle relaxation^#^	> 500			
Set airflows – Magills, circle, T-piece	1014			
Spinal anaesthetic	162			
Ventilate patient with mask and hand	1014			*

## Appendix 2

**Table:**

Supervised skills	More than 20 learning opportunities	More than 5 learning opportunities	Insufficient learning opportunities	Procedures that happen inside theatre and elsewhere
Appendicectomy	20			
Pleural biopsy			0	*
Hydrocoelectomy			3	
Vasectomy		9		*
Reduction of nose fracture			0	*
Inject keloids			0	*
Phenol ablation of ingrown toenail			2	*
Skin graft		6		
Laparotomy for ectopic pregnancy		16		
Termination of pregnancy (if no moral objection)	50			*
Relieve cardiac tamponade			1	*
Tracheostomy			2	*
Amputations – fingers			4	
Debridement of open fractures			1	*
Fasciotomy			0	
Biers block			0	
Brachial block			0	
Epidural anaesthesia			0	

## Appendix 3

**Table:**

Elective Skills	More than 20 learning opportunities	More than 5 learning opportunities	Insufficient learning opportunities	Procedures that happen inside theatre and elsewhere
Anal sphincterotomy			1	
Gastroscopy			0	*
Hernia repair	39			
Injection of haemorrhoids			1	
Rubber banding of haemorrhoids			0	
Anal dilatation			0	
Sigmoidoscopy			4	
Liver biopsy			0	*
Bilateral sub capsular orchidectomy		5		
Cystoscopy			0	
El Ghorab shunt for priapism			0	
Prostate biopsy		7		
Varicolectomy			0	
Orchidectomy and anchoring of torted testis			3	
Cataract removal	90			
Evisceration of eye			0	
Tonsillectomy and adenoidectomy	67			
Cervical cerclage for incompetence			3	
Cone biopsy of cervix			1	
Cervical polyp removal			1	
Colposcopy	117			
Hysterectomy	27			
Loop electrosurgical excision procedure for cervix	93			
Laparotomy for stabbed abdomen		19		
Laparotomy for bowel obstruction				
Burr holes			1	
Open reductions, pins and screws		6		
Repair nerves and tendons		15		
Dental extraction	134			*
Wiring of teeth for mandibular fractures			0	
